# Associations of genetically predicted *Helicobacter pylori* antibodies with aneurysmal subarachnoid hemorrhage and unruptured intracranial aneurysm: A two-sample mendelian randomization study

**DOI:** 10.1097/MD.0000000000051178

**Published:** 2026-10-16

**Authors:** Zhaohui Yi, Chengyun Wang, Qingyan Wu, Xiaobin Mao, Guohua Mao, Jicai Zhang

**Affiliations:** a Department of Neurosurgery, The Second Affiliated Hospital, Jiangxi Medical College, Nanchang University, Nanchang, China; b Jiangxi Vocational and Technical College of Communications, Nanchang, Jiangxi Province, China.

**Keywords:** Cerebral aneurysms, *Helicobacter pylori* antibodies, Mendelian randomization, Subarachnoid hemorrhage, Vacuolating cytotoxin-A

## Abstract

The causal relationship between *Helicobacter pylori* antibodies and the risk of cerebral aneurysms remains unclear. We used Mendelian randomization analysis to assess the causal relationship between *Helicobacter pylori* antibodies and the risk of cerebral aneurysms. A genome-wide association study (GWAS) dataset from the IEU Open GWAS project was utilized for antibodies to 7 different *Helicobacter pylori*-specific proteins, and from the Finnish database for cerebral aneurysms that included unruptured aneurysm (uIA) and aneurysmal subarachnoid hemorrhage (aSAH). Mendelian randomization (MR) analysis was used to evaluate the causal relationship between *Helicobacter pylori* antibodies and cerebral aneurysms. Multivariable Mendelian randomization was used to assess whether the VacA–aSAH association persisted after adjustment for systolic blood pressure, dietary magnesium intake, and dietary calcium intake. A Bonferroni-corrected significance threshold of *P* < .0036 was applied to the 14 primary comparisons. Genetically predicted serum antibody levels of *Helicobacter pylori* (*H. pylori*) vacuolating cytotoxin-A (VacA) showed a nominal inverse association with aSAH (odds ratio (OR) = 0.88, 95% CI: 0.78–0.99, *P* = .044), but were not associated with uIA. However, this association did not remain statistically significant after Bonferroni correction. Other *H. pylori* antibodies were not causally associated with aSAH and uIA. In the MVMR analyses, the VacA–aSAH association was no longer statistically significant after adjustment for systolic blood pressure or dietary calcium intake, whereas it remained nominally significant after adjustment for dietary magnesium intake. These findings indicate changes after adjustment but do not establish mediation. Our study suggests that increased levels of serum *Helicobacter pylori* vacuolating cytotoxin-A (VacA) antibodies may be inversely associated with aneurysmal subarachnoid hemorrhage (aSAH). The attenuation of the association after adjustment for systolic blood pressure and dietary calcium intake suggests that these factors may partly account for the observed association, although mediation cannot be confirmed. These preliminary findings require external validation in independent populations and larger GWAS datasets before clinical implications can be considered.

## 1. Introduction

Intracranial aneurysms are focal dilations of cerebral arteries. Their rupture can cause aneurysmal subarachnoid hemorrhage (aSAH), which is associated with substantial mortality and disability.^[1–5]^ Understanding factors associated with aneurysm susceptibility may help clarify the biological processes underlying these outcomes.

*Helicobacter pylori* (*H. pylori*) is a Gram-negative, spiral-shaped bacterium distributed worldwide. *H. pylori* can cause chronic gastritis and peptic ulcers.^[6]^ *H. pylori* antibody traits include immunoglobulin G (IgG), GroEL, outer membrane protein (OMP), urease subunit-A (UreA), vacuolating cytotoxin-A (VacA), cytotoxin-associated gene-A (CagA), and catalase antibodies.^[7]^

Previous Mendelian randomization (MR) studies have examined *H. pylori* antibody traits in relation to stroke and atherosclerosis.^[8,9]^ These studies provide a vascular context but do not directly resolve the antibody-specific associations with intracranial aneurysm outcomes. A broader MR study of digestive diseases included aneurysm and subarachnoid hemorrhage outcomes, but evaluated disease susceptibility rather than the same antibody traits.^[10]^

The objective of this study was to examine the associations of seven genetically predicted *H. pylori* antibody traits with aSAH and unruptured intracranial aneurysm (uIA), and to assess whether the VacA association differed after accounting for vascular and mineral traits.

## 2. Materials and methods

### 2.1. Study design and data sources

A two-sample MR design was used to examine *H. pylori* antibody traits in relation to the 2 aneurysm outcomes.^[11]^ Single-variable MR (SVMR) was followed by multivariable MR (MVMR) models incorporating systolic blood pressure, dietary magnesium intake, or dietary calcium intake. Single nucleotide polymorphisms (SNPs) associated with the antibody traits were obtained from genome-wide association studies (GWAS) available through the Integrative Epidemiology Unit (IEU) OpenGWAS resource.

In this MR study, pooled GWAS data for 7 *H. pylori* antibodies, aSAH and uIA, systolic blood pressure, dietary magnesium intake, and dietary calcium intake in individuals of European ancestry were used. The *H. pylori* protein antibodies GWAS dataset from the UK Biobank included up to 8735 individuals with anti-*Helicobacter pylori* antibody levels, including *Helicobacter pylori* IgG, GroEL, OMP, UREA, CagA, VacA, and catalase.^[7]^ We used SNPs associated with *H. pylori* antibodies as instrumental variables (IVs). We selected genetic association data related to cerebral aneurysms that included uIA and aSAH from the Finnish database. The cerebral aneurysms database can be accessed online through the IEU Open GWAS project website. The dataset for aSAH included 2127 cases and 203,068 controls of European ancestry, with the accession number finn-b-I9_SAHANEUR. For uIA, the dataset included 992 cases and 203,068 controls of European ancestry, with the accession number finn-b-I9_ANEURYSM.^[12]^ The GWAS summary statistics for systolic blood pressure, dietary magnesium intake, and dietary calcium intake were obtained from the IEU OpenGWAS database, with accession numbers ebi-a-GCST90000062, ukb-b-7372, and ukb-b-8951, respectively. The systolic blood pressure dataset included 810,865 individuals of European ancestry, whereas the dietary magnesium intake and dietary calcium intake datasets each included 64,979 European participants. Detailed characteristics and source information for all GWAS datasets are provided in Table S8, Supplemental Digital Content 1. Although detailed demographic variables (such as age and gender) of the samples are not directly provided in the database, we conducted analyses based on summary statistical data related to genetic variations and phenotype associations, including p-values, effect sizes, and standard errors provided by the database. All summary data used in this study were obtained from the Medical Research Council Integrative Epidemiology Unit (MRC-IEU) OpenGWAS unified archive (https://gwas.mrcieu.ac.uk/).

### 2.2. Standard protocol approval, registration, and patient consent

All data used in this MR analysis were publicly available summary-level GWAS data. Therefore, separate ethical approval and informed consent were not required for the present study. Ethical approval and informed consent had been obtained in the original GWAS studies.

### 2.3. Mendelian randomization analysis

The SNPs from GWAS were applied as IVs. An instrument-selection threshold of *P* < 5 × 10^−6^ was applied because the conventional genome-wide significance threshold of *P* < 5 × 10^−8^ yielded too few instruments for several antibody traits.^[13]^ To evaluate the potential influence of weak instruments, the proportion of variance explained (R^2^) and F-statistics were calculated, with an F-statistic > 10 considered indicative of adequate instrument strength. The instrument-strength statistics for the seven antibody traits are presented in Table S2, Supplemental Digital Content 2.

The IVs were clumped according to the linkage disequilibrium (LD) structure of European ancestry (r^2^ < 0.001 within 10,000 kb). Exposure and outcome data were initially matched using rsIDs, with position-based matching attempted when rsID matching was unavailable. SNPs that were absent from the outcome datasets and palindromic SNPs with ambiguous allele frequencies were excluded. The detailed harmonization and quality-control workflow is provided in Table S7, Supplemental Digital Content 3. Causal estimates were evaluated in R using the TwoSampleMR software package (V.0.5.7), using inverse variance weighting (IVW) as the primary analysis method.^[14]^ The weighted median, MR-Egger, simple mode, and weighted mode methods were used as complementary analyses. For MVMR, three separate models were constructed by combining the IVs for VacA antibody levels with those for systolic blood pressure, dietary magnesium intake, or dietary calcium intake, respectively, using aSAH as the outcome.^[14–17]^ The combined instruments were clumped and harmonized using the same criteria as those applied in the SVMR analyses. The MVMR estimates were interpreted as associations after mutual adjustment rather than as definitive evidence of mediation. The detailed MVMR results are presented in Table S3, Supplemental Digital Content 4.

Because seven antibody traits were evaluated against 2 aneurysm outcomes, a Bonferroni-corrected significance threshold of *P* < .0036 (0.05/14) was applied to the 14 primary IVW comparisons. Associations with *P* values between .0036 and .05 were considered nominally significant. The complete multiple-testing results are presented in Table S1, Supplemental Digital Content 5. Minimum detectable odds ratios at 80% statistical power for the 14 primary antibody–outcome comparisons are provided in Table S6, Supplemental Digital Content 6.

### 2.4. Heterogeneity and sensitivity analyses

To evaluate instrument strength, the F-statistic was calculated, and an F-value > 10 was considered to indicate a low likelihood of weak-instrument bias.^[8]^ Cochran’s Q test was used to assess heterogeneity among the SNP-specific causal estimates, with *P* < .05 indicating evidence of heterogeneity. The MR-Pleiotropy RESidual Sum and Outlier (MR-PRESSO) global and outlier tests and the MR-Egger intercept test were applied to assess potential horizontal pleiotropy. A *P*-value < .05 for the MR-Egger intercept or MR-PRESSO global test was considered evidence of horizontal pleiotropy. When MR-PRESSO identified potential outlier SNPs, the outlier-corrected estimate and distortion test were examined where available. Leave-one-out analysis was also performed to determine whether the overall estimate was disproportionately influenced by any single SNP.^[18]^ Detailed heterogeneity and MR-Egger intercept results are provided in Table S4, Supplemental Digital Content 7, and the MR-PRESSO results are presented in Table S5, Supplemental Digital Content 8.

## 3. Results

### 3.1. Genetic instruments

At the instrument-selection threshold of *P* < 5 × 10^−6^, 10, 10, 9, 5, 10, 10, and 15 candidate IVs were identified for anti-*H. pylori* IgG, CagA, catalase, GroEL, OMP, UreA, and VacA antibody levels, respectively. After matching with the outcome datasets and harmonization, 10, 10, 8, 3, 8, 10, and 13 SNPs remained for the corresponding antibody traits. Five SNPs were unavailable in the outcome datasets, and 2 ambiguous palindromic SNPs were excluded. The instrument-selection and harmonization workflow is presented in Table S7, Supplemental Digital Content 3.

The minimum F-statistic across all candidate instruments was 20.87, and the mean F-statistics for the seven antibody traits ranged from 22.48 to 24.38, indicating that all individual instruments had F-statistics >10. The corresponding approximate R^2^ values and instrument-strength statistics are presented in Table S2, Supplemental Digital Content 2.

### 3.2. Associations with aneurysm outcomes

Using the IVW method, genetically predicted serum *H. pylori* VacA antibody levels showed a nominal inverse association with the risk of aSAH (OR = 0.88, 95% CI: 0.78–0.99, raw *P* = .044). However, this association did not remain statistically significant after Bonferroni correction for the 14 primary comparisons (adjusted *P* = .616; Table S1, Supplemental Digital Content 5). The weighted median estimate showed a direction consistent with that of the IVW estimate (Fig. 1). Leave-one-out analysis showed that no single SNP disproportionately influenced the VacA–aSAH estimate (Fig. 2). Reverse MR analysis did not provide evidence of a causal effect of aSAH on VacA antibody levels. There was no association between VacA antibody levels and uIA (OR = 0.94, 95% CI: 0.78–1.12, *P* = .475). *H. pylori* IgG, GroEL, OMP, UreA, CagA, and catalase antibody levels were not associated with aSAH or uIA (Fig. 3). None of the 14 primary antibody–outcome comparisons reached the Bonferroni-corrected significance threshold of *P* < .0036.

**Figure 1.**
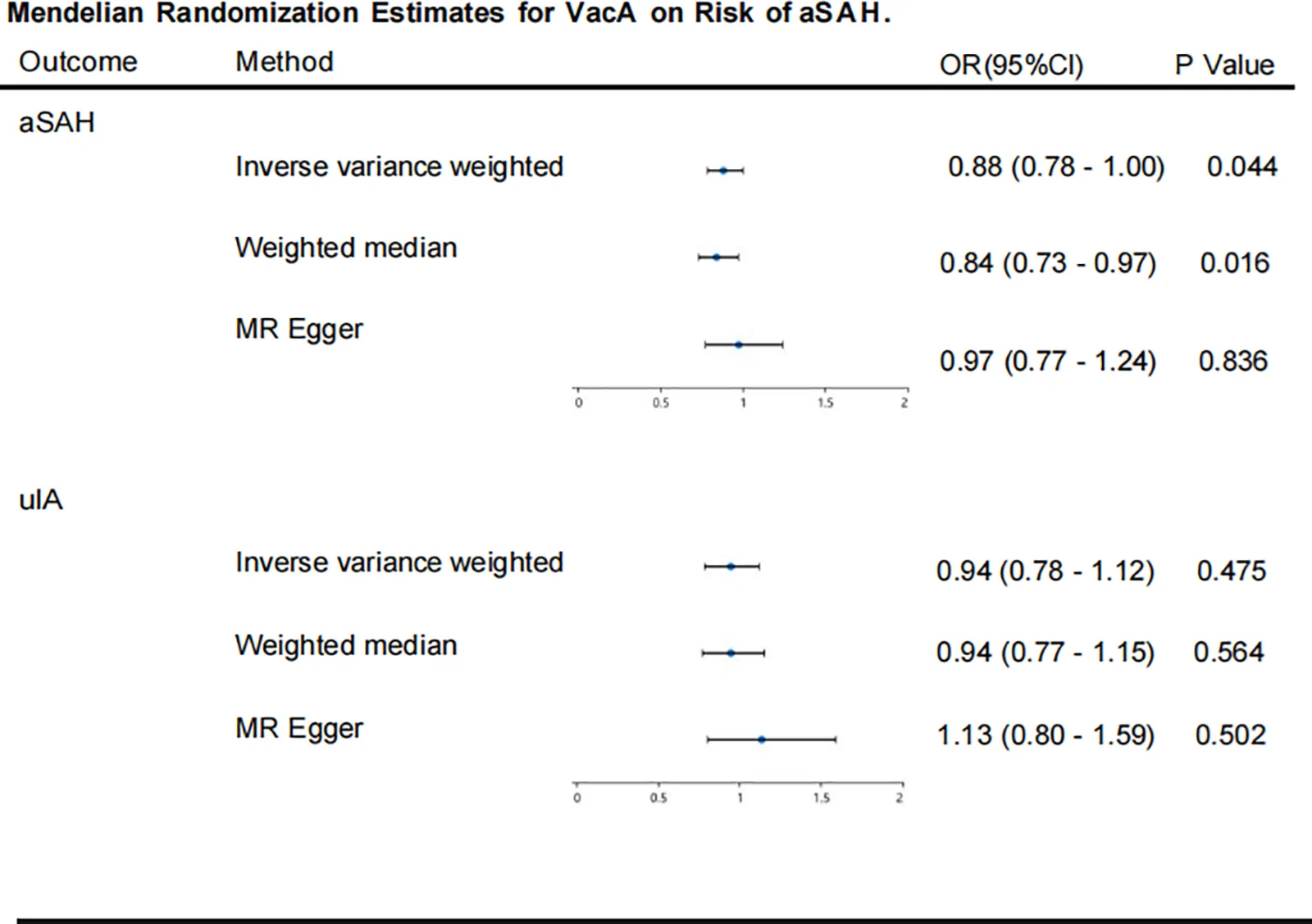
Mendelian randomization estimates for the associations of genetically predicted *Helicobacter pylori* vacuolating cytotoxin-A antibody levels with aneurysmal subarachnoid hemorrhage and unruptured intracranial aneurysm. Effect estimates are presented as odds ratios with 95% confidence intervals using the inverse-variance weighted, weighted median, and MR-Egger methods. The inverse-variance weighted estimate showed a nominal inverse association between vacuolating cytotoxin-A antibody levels and aneurysmal subarachnoid hemorrhage, although this association did not remain statistically significant after Bonferroni correction. aSAH = aneurysmal subarachnoid hemorrhage, CI = confidence interval, IVW = inverse-variance weighted, MR = Mendelian randomization, OR = odds ratio, uIA = unruptured intracranial aneurysm, VacA = vacuolating cytotoxin-A.

**Figure 2.**
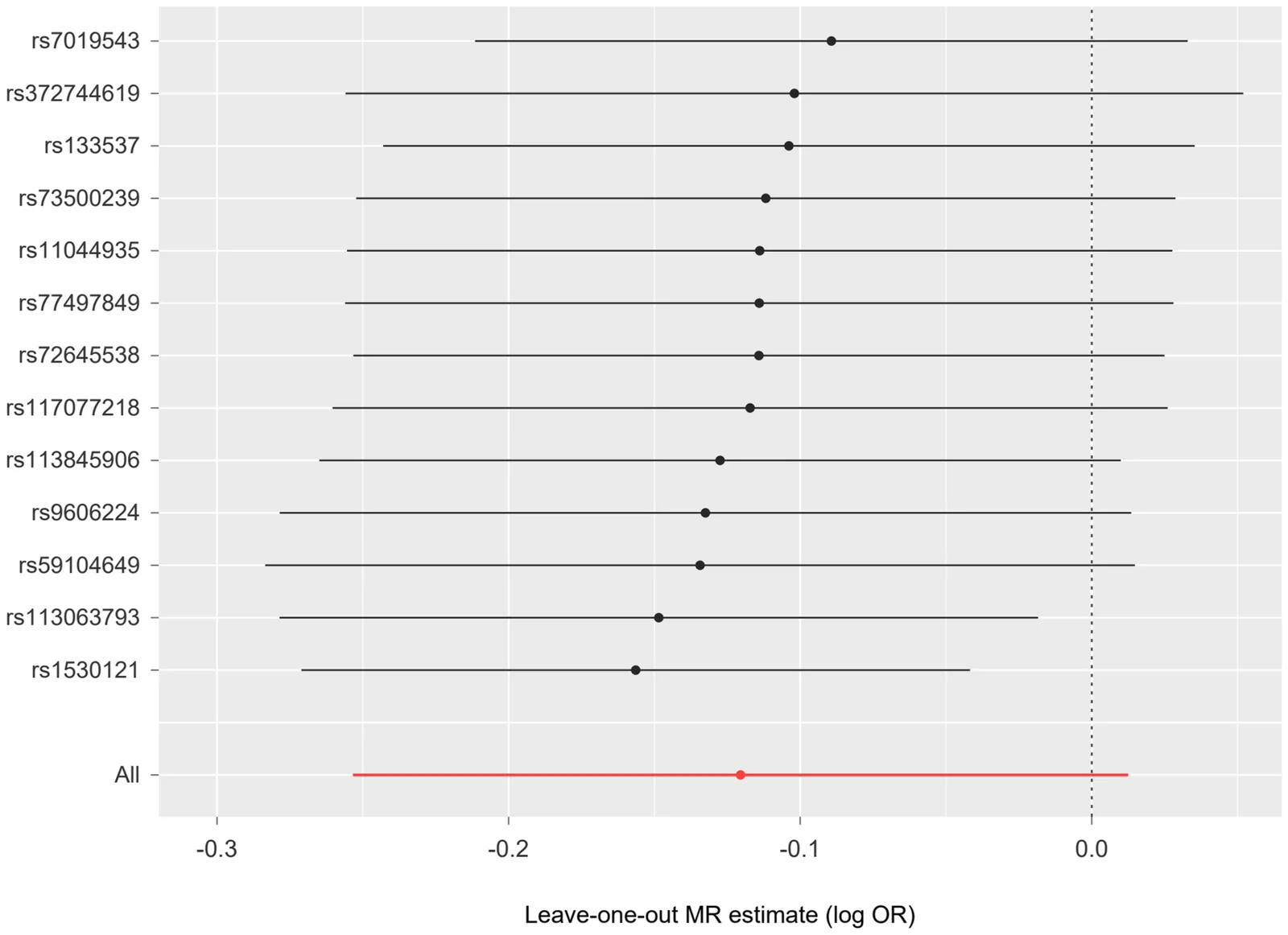
Leave-one-out sensitivity analysis of the association between genetically predicted *Helicobacter pylori* vacuolating cytotoxin-A antibody levels and aneurysmal subarachnoid hemorrhage. Each genetic instrument was sequentially excluded, and the inverse-variance weighted estimate was recalculated using the remaining instruments. No single genetic instrument disproportionately influenced the overall estimate. aSAH = aneurysmal subarachnoid hemorrhage, CI = confidence interval, IVW = inverse-variance weighted, MR = Mendelian randomization, SNP = single nucleotide polymorphism, VacA = vacuolating cytotoxin-A.

**Figure 3.**
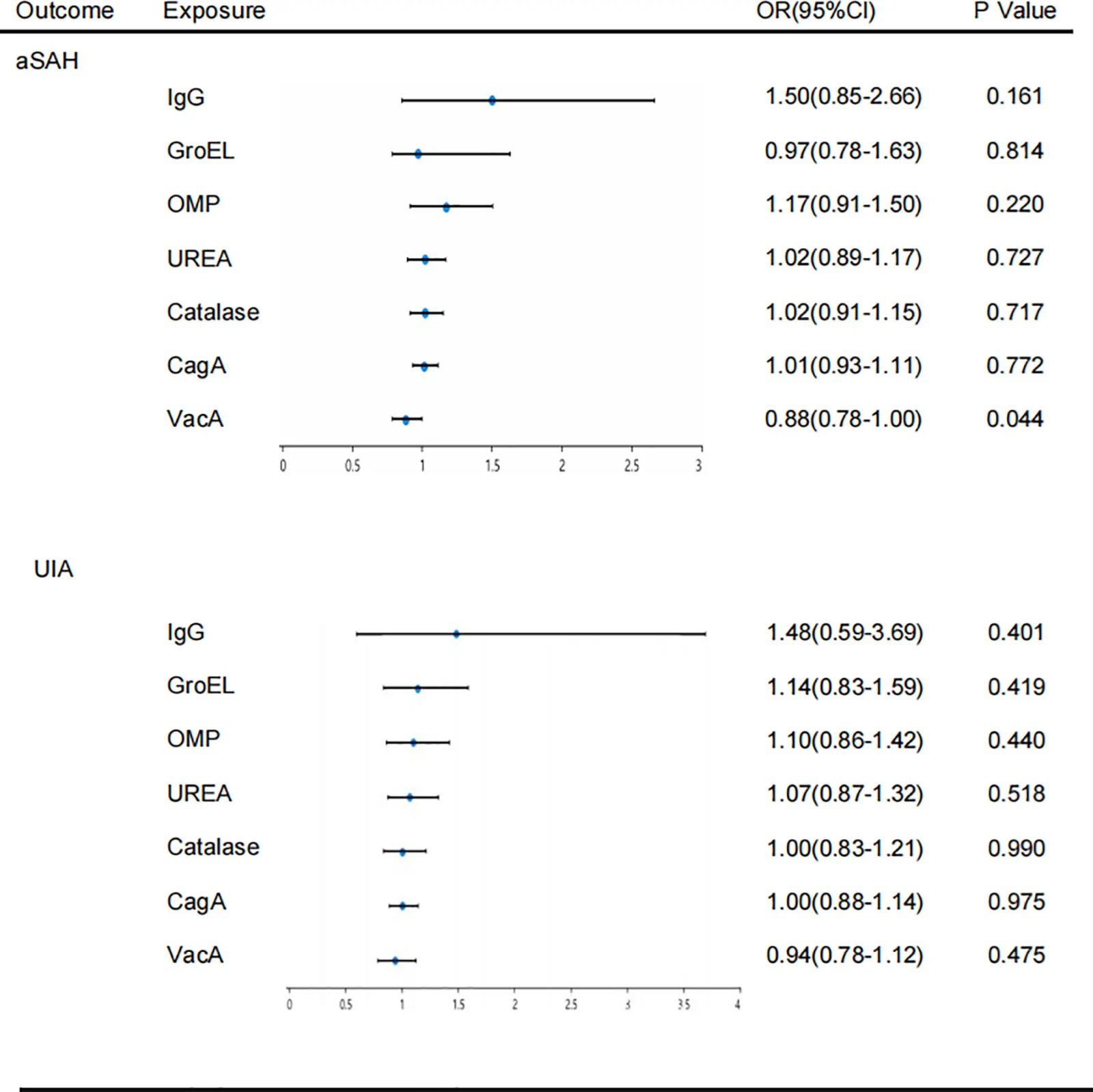
Mendelian randomization estimates for the associations of seven genetically predicted *Helicobacter pylori* antibody traits with aneurysmal subarachnoid hemorrhage and unruptured intracranial aneurysm. Effect estimates are presented as odds ratios with 95% confidence intervals. None of the 14 primary antibody–outcome comparisons reached the Bonferroni-corrected significance threshold of *P* < .0036. The evaluated antibody traits included *H. pylori* immunoglobulin G, cytotoxin-associated gene-A, catalase, companion protein GroEL, outer membrane protein, urease subunit-A, and vacuolating cytotoxin-A. aSAH = aneurysmal subarachnoid hemorrhage, CagA = cytotoxin-associated gene-A, CI = confidence interval, GroEL = companion protein GroEL, IgG = immunoglobulin G, IVW = inverse-variance weighted, OMP = outer membrane protein, OR = odds ratio, uIA = unruptured intracranial aneurysm, UreA = urease subunit-A, VacA = vacuolating cytotoxin-A.

### 3.3. Heterogeneity and sensitivity analyses

Cochran’s Q test indicated heterogeneity in the IVW estimates for OMP antibodies and aSAH (Q = 14.42, df = 7, *P* = .044), VacA antibodies and aSAH (Q = 22.81, df = 12, *P* = .029), and VacA antibodies and uIA (Q = 23.13, df = 12, *P* = .027). No MR-Egger intercept test provided evidence of directional horizontal pleiotropy; for the VacA–aSAH association, the intercept was − 0.0247 (*P* = .352). Detailed heterogeneity and MR-Egger results are presented in Table S4, Supplemental Digital Content 7.

The MR-PRESSO global test was significant for the VacA–aSAH analysis (*P* = .0466) and identified rs1530121 as a potential outlier. However, the distortion test was not statistically significant (*P* = .294). The MR-PRESSO global test was also significant for the VacA–uIA analysis (*P* = .036), although no individual outlier SNP was identified. The complete MR-PRESSO results are presented in Table S5, Supplemental Digital Content 8.

### 3.4. Multivariable Mendelian randomization analysis

The mutually adjusted VacA estimates are presented in Table S3, Supplemental Digital Content 4 and Figure 4. After adjustment for systolic blood pressure, the estimate was OR = 0.99 (95% CI: 0.90–1.09, *P* = .911). After adjustment for dietary calcium intake, the estimate was OR = 1.05 (95% CI: 0.95–1.16, *P* = .318). After adjustment for dietary magnesium intake, the estimate was OR = 0.88 (95% CI: 0.79–0.99, *P* = .027).

**Figure 4.**
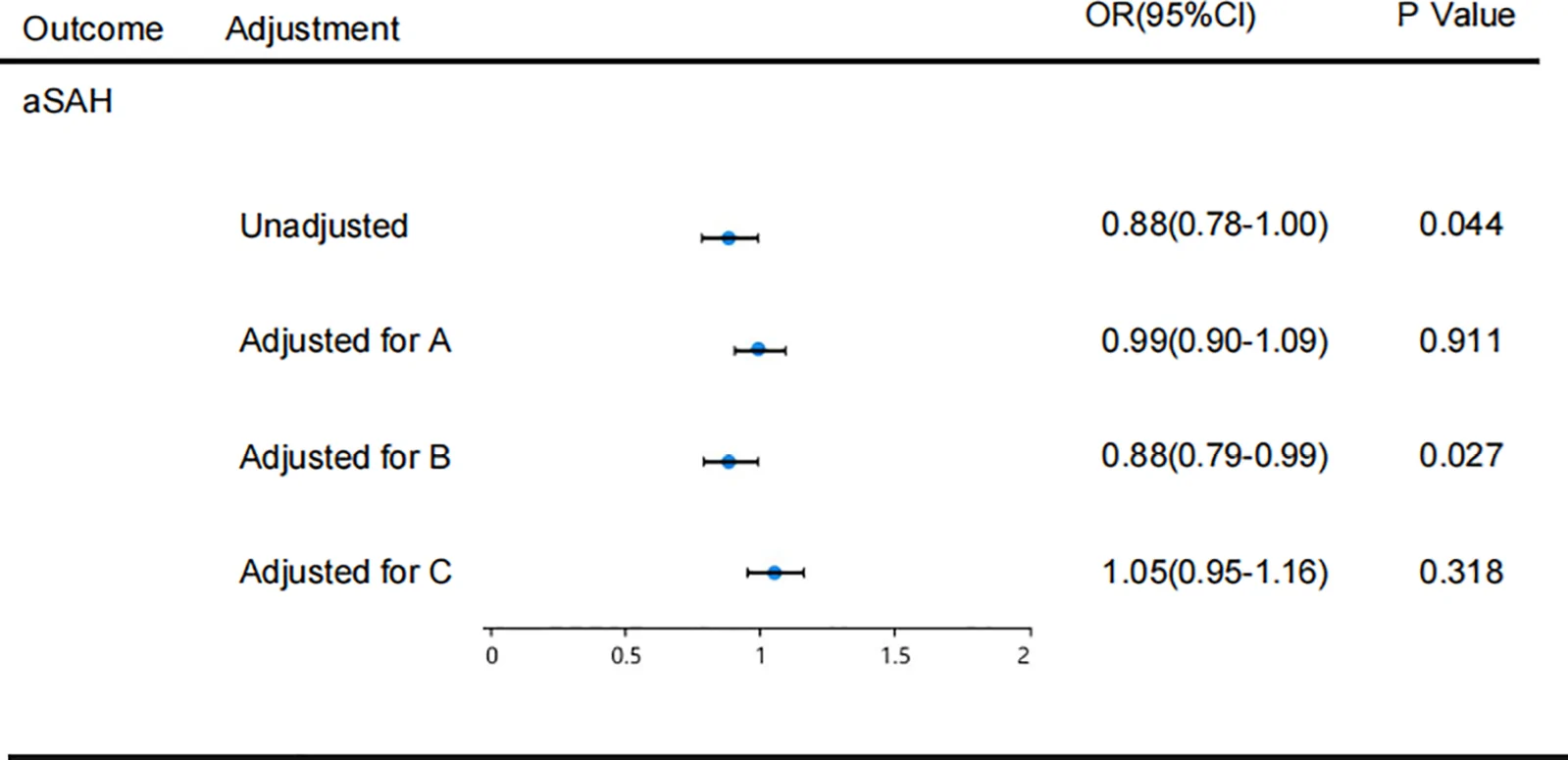
Multivariable Mendelian randomization estimates for the association between genetically predicted *Helicobacter pylori* vacuolating cytotoxin-A antibody levels and aneurysmal subarachnoid hemorrhage after adjustment for vascular and mineral traits. The panels present mutually adjusted estimates for vacuolating cytotoxin-A antibody levels after adjustment for (A) systolic blood pressure, (B) dietary magnesium intake, and (C) dietary calcium intake. Effect estimates are presented as odds ratios with 95% confidence intervals. These results represent mutually adjusted associations and should not be interpreted as definitive evidence of mediation. aSAH = aneurysmal subarachnoid hemorrhage, CI = confidence interval, MVMR = multivariable Mendelian randomization, OR = odds ratio, SBP = systolic blood pressure, VacA = vacuolating cytotoxin-A.

## 4. Discussion

### 4.1. Principal findings

None of the primary antibody–outcome associations met the multiple-testing threshold. The exploratory inverse VacA estimate therefore does not establish a protective effect. The study addresses whether antibody-specific responses are associated with aneurysm outcomes, with interpretation depending on the outcome definitions, instrument selection, and precision of the estimates.

### 4.2. Comparison with previous studies

Guo et al examined the same spectrum of *H. pylori* antibody traits and reported positive associations of genetically predicted VacA antibody levels with all-cause and cardioembolic stroke.^[8]^ The inverse direction reported here differs from those findings. However, broad stroke categories and aneurysm-related outcomes represent different clinical questions. Differences in outcome ascertainment, sample composition, and statistical uncertainty may contribute to the observed differences.

Similarly, Zhang et al reported a positive association of VacA antibody levels with coronary atherosclerosis and of OMP antibody levels with peripheral atherosclerosis.^[9]^ Their findings concern atherosclerotic disease rather than aneurysm rupture. These comparisons support examining individual antibody traits in relation to defined vascular outcomes instead of treating *H. pylori* serology as a uniform exposure.

Qin et al investigated digestive disease susceptibility across cerebrovascular outcomes, including intracranial aneurysm and subarachnoid hemorrhage, and reported associations involving gastroesophageal reflux disease.^[10]^ Their exposure definition differs from the antibody traits evaluated here. The distinction of the present analysis is therefore its antibody-specific question, rather than a general claim that digestive factors have not previously been studied in relation to aneurysms.

### 4.3. Biological and clinical interpretation

Circulating VacA antibodies reflect the host immune response to *H. pylori* and cannot be equated with the concentration or activity of VacA toxin.^[7]^ Experimental work has examined VacA-related disruption of calcium signaling in T lymphocytes,^[19]^ while broader cellular studies describe effects on vacuolation, intracellular trafficking, and cell survival.^[20]^ These observations provide biological context, but do not demonstrate that a stronger antibody response protects the cerebral arterial wall. The present genetic estimates cannot distinguish an effect of the antibody response from other features of infection or host immunity.

Accordingly, the findings do not support using VacA antibody levels to guide aneurysm screening or treatment. A relevant clinical question for future studies is whether a reproducible antibody association adds information beyond established vascular risk factors, with aneurysm presence and subsequent rupture evaluated separately.

### 4.4. Interpretation of the multivariable analyses

The changes in the VacA estimates across the multivariable models should be interpreted as differences after mutual adjustment. Attenuation can arise from correlated traits, shared genetic pathways, differences in instrument composition, or imprecise conditional estimates; loss of statistical significance alone does not identify a mediator. Likewise, an association remaining after adjustment for one trait does not rule out a biological role for that trait.

A mechanistic interpretation would require reliable instruments for each exposure, assessment of conditional instrument strength, and clearly specified direct and indirect effects. Dedicated mediation analyses would be a subsequent research step rather than a conclusion from the current adjustment models.

### 4.5. Strengths and limitations

The principal strength of this study is the use of an MR framework to evaluate seven *H. pylori* antibody traits in relation to aSAH and uIA as distinct intracranial aneurysm outcomes. Compared with conventional observational analyses, MR may reduce, although not completely eliminate, bias due to confounding and reverse causation. In addition, multiple MR estimators, leave-one-out analysis, heterogeneity testing, MR-Egger intercept testing, MR-PRESSO analysis, and correction for multiple comparisons were used to evaluate the robustness of the findings.

The relaxed instrument-selection threshold may admit less specific genetic associations, even when individual F-statistics exceed the conventional screening threshold. Single-variable instrument strength also does not establish adequate conditional strength in multivariable models.

The nominal VacA association did not survive multiple-testing correction. Differences in the variance explained by the instruments and the limited numbers of outcome cases restrict precision across the antibody–outcome comparisons. A nonsignificant result therefore should not be interpreted as excluding all clinically relevant effects.

Evidence of heterogeneity in several analyses raises the possibility that the instruments capture more than one biological pathway. A nonsignificant MR-Egger intercept does not exclude horizontal pleiotropy, particularly when few instruments are available. The sensitivity analyses should consequently be considered together rather than using a single test as proof of robustness.

The antibody GWAS datasets had relatively modest sample sizes, and VacA antibody levels represent genetically predicted host immune responses rather than direct measurements of VacA toxin exposure or biological activity. Consequently, the biological interpretation of the observed association remains uncertain.

Finally, all analyses were based predominantly on participants of European ancestry. The generalizability of these findings to other ancestral populations remains unknown.

### 4.6. Future research

Replication should use larger antibody GWAS and independent cohorts with adjudicated aneurysm outcomes, separating aneurysm presence from incident rupture. Analyses in populations of diverse ancestry would help assess transferability. Prospective studies could measure infection status, antibody profiles, and vascular risk factors before the outcome, allowing their temporal relationships to be examined.

Where suitable instruments are available, conditional-strength assessment and colocalization could clarify whether genetic signals are shared, before dedicated mediation analyses are attempted. Experimental studies should also distinguish the effects of VacA toxin from those of the antibody response in vascular and immune-cell systems.

## Author contributions

**Conceptualization:** Zhaohui Yi, Guohua Mao.

**Formal analysis:** Zhaohui Yi, Chengyun Wang.

**Writing – original draft:** Zhaohui Yi.

**Methodology:** Chengyun Wang.

**Data curation:** Qingyan Wu, Xiaobin Mao.

**Visualization:** Qingyan Wu.

**Validation:** Xiaobin Mao.

**Supervision:** Guohua Mao, Jicai Zhang.

**Project administration:** Jicai Zhang.

**Writing – review & editing:** Jicai Zhang.
















